# Novel pediatric granulomatosis with polyangiitis with a marked bloody pericardial effusion and bloody stool: a case report

**DOI:** 10.1186/s13223-021-00627-1

**Published:** 2021-12-04

**Authors:** Memi Kato, Keisuke Jimbo, Masumi Nagata, Yoshiko Endo, Kosuke Kashiwagi, Kimiko Maruyama, Natsuki Ito, Kaori Tokushima, Nobuyasu Arai, Reiko Kyodo, Masamichi Sato, Eri Miyata, Kenji Hosoi, Eisuke Inage, Tamaki Ikuse, Hideo Fukunaga, Takahiro Kudo, Toshiaki Shimizu

**Affiliations:** Department of Pediatrics, Juntendo University, Faculty of Medicine, 2-1-1 Hongo, Bunkyo-ku, Tokyo, 113-8421 Japan

**Keywords:** Bloody stool, Cardiac tamponade, Chest pain, Granulomatosis with polyangiitis, Pericardial effusion, Serine proteinase 3-anti-neutrophil cytoplasmic antibody, Vasculitis

## Abstract

**Background:**

Granulomatosis with polyangiitis (GPA) is a syndrome of refractory vasculitis involving the upper respiratory tract, lungs, kidneys, and systemic small and medium-sized arteries that affects all age groups. No pediatric case with a bloody pericardial effusion resulting in cardiac tamponade and co-existing hematochezia has been reported.

**Case presentation:**

A 14-year-old boy was referred for evaluation of prolonged fever, chest pain, and intermittent hematochezia. Diagnostic imaging showed a prominent pericardial effusion. Immediately after admission, his systolic blood pressure decreased. Emergent pericardiocentesis resulted in aspiration of a massive amount of bloody pericardial fluid. This was diagnosed as cardiac tamponade because his blood pressure recovered immediately after the drainage. The patient had an elevated serine proteinase 3-anti-neutrophil cytoplasmic antibody (PR3-ANCA) level on serological examination. Head MRI showed thickening of nasal and sinusoidal mucosa and a cystic mass in the left sphenoid sinus. After ruling out malignancy based on the cytology of the effusion, chest MRI, and gallium scintigraphy, total colonoscopy showed multiple irregular-shaped aphthae from the right transverse colon to the cecum on the contralateral side of the mesenteric attachments. Biopsy specimens of aphthous lesions confirmed necrotizing granulomatous inflammation. A diagnosis of GPA was made based on these findings, and oral prednisolone (PSL) and azathioprine were started. The hematochezia disappeared rapidly, and no recurrence of pericardial effusion was seen after PSL tapering was completed. The PR3-ANCA level decreased into the normal range immediately after the initial therapy.

**Conclusions:**

Pericarditis is a common cardiac complication of GPA, but there have been no reports of resultant cardiac tamponade. This is the first case of pediatric GPA with cardiac and gastrointestinal complications preceding the common symptoms such as respiratory or renal symptoms. A case of pediatric GPA with hematochezia is also extremely rare. In conclusion, serial measurement of ANCA levels is important in patients with persistent fever and bloody stool, such as in inflammatory bowel disease, to make the diagnosis of a vasculitic syndrome.

**Supplementary Information:**

The online version contains supplementary material available at 10.1186/s13223-021-00627-1.

## Background

Granulomatosis with polyangiitis (GPA) is a vasculitic syndrome, formerly called Wegener’s granulomatosis, and was re-classified as GPA at the 2012 Chapel Hill Consensus Conference (CHCC 2012). The three main manifestations of GPA are: (1) necrotizing granulomatous inflammation involving the upper and lower respiratory tracts; (2) necrotizing glomerulonephritis; and (3) systemic necrotizing granulomatous vasculitis affecting predominantly small to medium vessels, in which anti-neutrophil cytoplasmic antibody (ANCA) is involved [1]. Although the clinical course of pediatric GPA is somewhat similar to that in adults [2], adult cases with gastrointestinal complications such as inflammatory bowel disease [3, 4] and cardiac diseases [5, 6] have been reported.

A case of pediatric GPA in which hematochezia and diarrhea as gastrointestinal symptoms and pericarditis as a cardiac complication preceded the main symptoms is presented. In this case, the GPA resulted in secondary cardiac tamponade due to pericarditis. A literature survey did not identify similar cases in the adult population. In addition, there were no previous reports of pediatric cases with gastrointestinal involvement preceding the main symptoms.

## Case presentation

A 14-year-old boy with no specific medical or family history was referred to our institute in order to identify the origin of his fever. 2 months before admission, he had a small amount of intermittent hematochezia (once or twice a day at most) attached to soft stools. Furthermore, he suffered from remittent fever of around 39 °C and malaise followed by diarrhea and appetite loss 3 weeks before admission. Thus, he visited his family physician and was referred to our institute for further investigation. However, he developed chronic chest pain before the outpatient follow-up (1 week before admission). Transthoracic echocardiography and thoracoabdominal contrast-enhanced CT showed a pericardial effusion. Because the pericardial effusion continued to increase, he was admitted to our institute for detailed examination and treatment of the underlying disease.

**Fig. 1:**
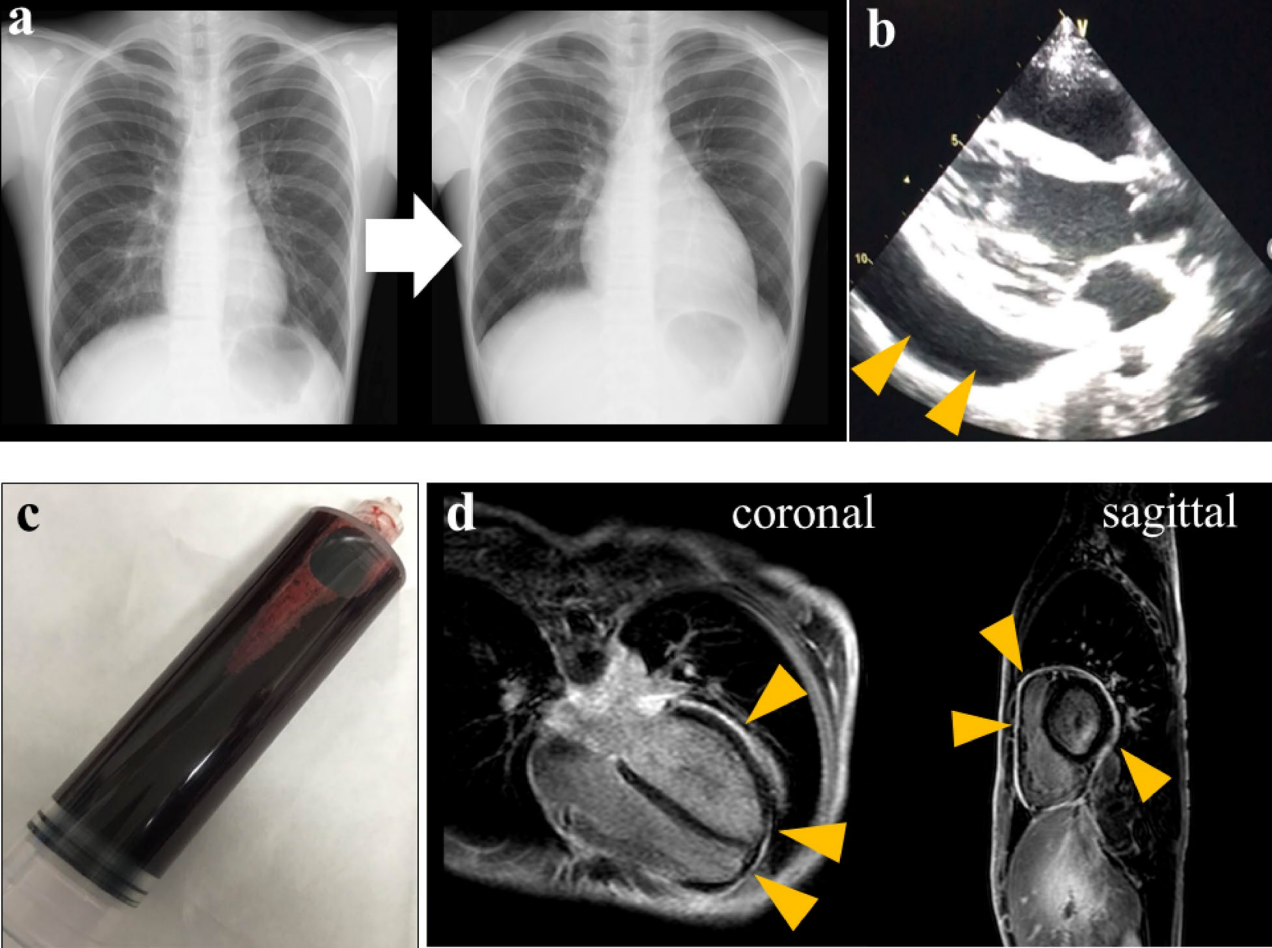
Findings of cardiovascular complications: **a** Cardiothoracic ratio on chest X-ray changes from 41.9% to 55.0% in a week. **b** Echocardiography shows a pericardial effusion (arrowhead). **c** Aspirated content by pericardiocentesis shows the bloody pericardial effusion. **d** Cardiac MRI shows contrast-enhanced delay consistent with the pericardium (arrowhead), suggestive of pericarditis

**Fig. 2:**
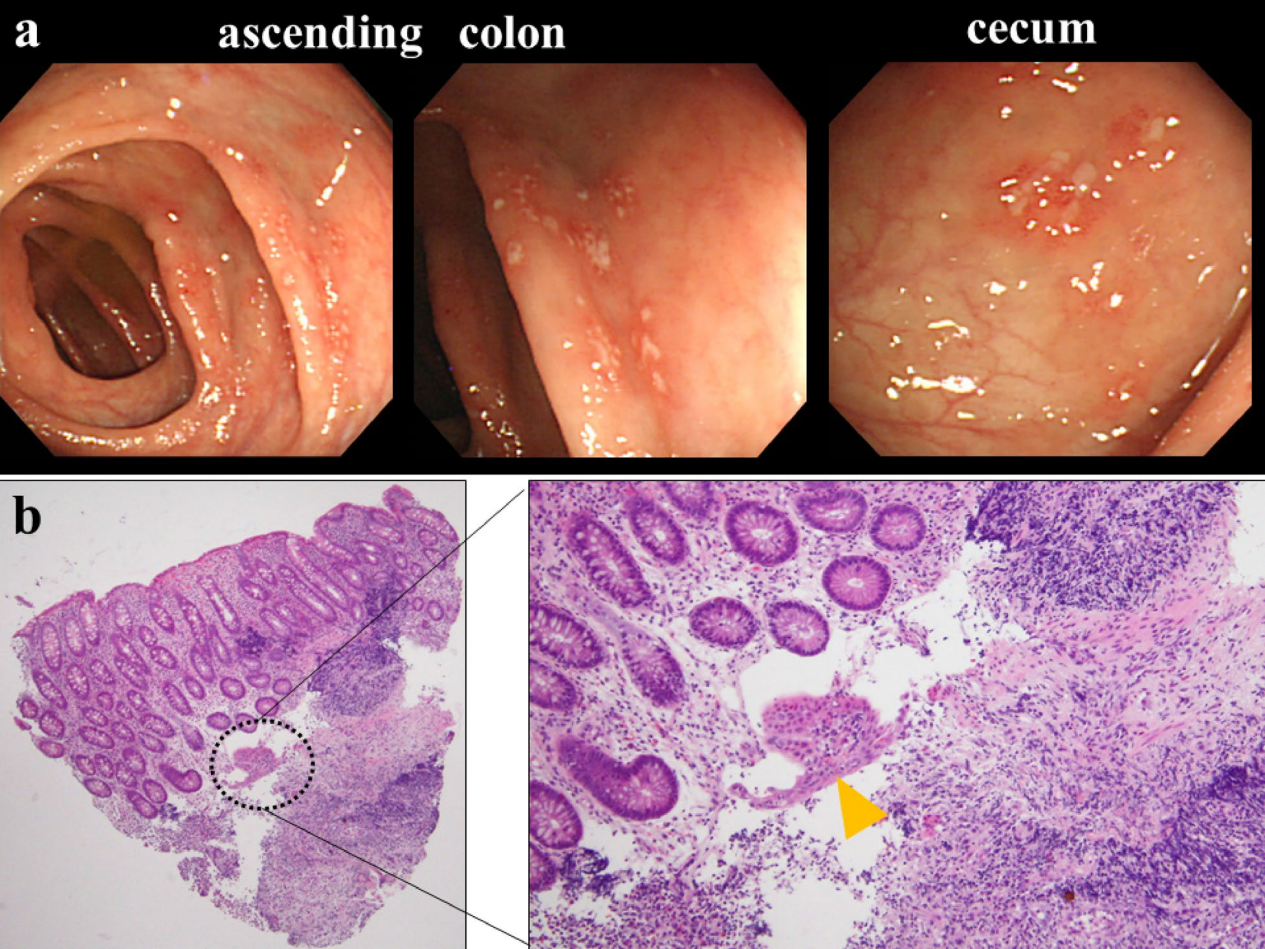
Findings of colonoscopy and histopathological findings of the colonic biopsy **a** Colonoscopy shows multiple irregular-shaped aphthae on the opposite side of the mesenteric attachment site from the cecum to the right transverse colon. **b** Colonic biopsy shows nonspecific infiltration to the colonic submucosa, and some granulomatous findings with prominent neutrophil infiltration adjacent to blood vessels (arrowhead)

At the time of admission, his height and weight were 170 cm (70th percentile) and 47.2 kg (20th percentile, 0.8 kg less than 2 months before admission), respectively. Body temperature, blood pressure, heart rate, respiratory rate, and oxygen saturation on room air were 37.1 °C, 116/61 mmHg, 110 beats/minute, 14 breaths/minute, and 97%, respectively. On physical examination, he had a mildly pale complexion and eyelid conjunctiva, diminished heart sounds, anterior chest pain relieved by bending forward, and mild tenderness in the lower mid-abdomen. The patient’s laboratory findings are summarized in Table 1. No remarkable abnormalities were found on urinalysis. An electrocardiogram showed decreased T wave amplitude in V1 to V3, which may be a finding of low potentials due to a pericardial effusion and myocarditis. On chest X-ray, the cardiothoracic ratio increased from 41.0% to 55.0% in the week before admission (Fig. 1a). Transthoracic echocardiography showed an echo-free space of 30 mm from the apex to the pericardium on the left ventricular axial view, indicating a pericardial effusion (Fig. 1b). In addition, anomalous motion of the ventricular septum was also confirmed, and the diameter of the inferior vena cava was 22 mm, with no respiratory variability. After admission, a sudden drop of blood pressure to 85/70 mmHg with tachycardia (132 beats/minute) appeared on the second day. Emergent pericardiocentesis was performed to prevent hemodynamic collapse; 500 mL of bloody pericardial fluid were aspirated by the puncture (Fig. 1c). His blood pressure recovered rapidly to 115/65 mmHg. The heart rate also decreased to 80 beats/minute immediately after the puncture. Thus, after the procedure, he was finally diagnosed as having cardiac tamponade. The aspirated fluid was an exudative pericardial fluid, and no malignant cells were found on cytological examination. No mediastinal tumor was identified on chest contrast-enhanced MRI, but contrast delay along the pericardium was observed, which was consistent with the pericarditis (Fig. 1d). Whole-body gallium scintigraphy also showed no abnormal accumulation. After pericardiocentesis, the inflammatory response decreased mildly, but the hematochezia persisted. To clarify the etiology, esophagogastroduodenoscopy (EGD) and colonoscopy (CS) were performed. EGD showed no macroscopic or histological abnormalities. In contrast, CS showed multiple irregular-shaped aphthae on the contralateral side of the mesenteric attachments from the right transverse colon to the cecum (Fig. 2a). No abnormalities were observed on small intestinal capsule endoscopy. Mucosal biopsy at the ascending colon and cecum showed nonspecific inflammatory cell infiltration into the submucosa and some granulomatous findings with prominent neutrophilic infiltration adjacent to blood vessels (Fig. 2b). Head MRI showed nasal and sinus mucosal thickening and a cystic mass in the left sphenoid sinus (Additional file 2: Fig. S1). No abnormalities were found on renal biopsy. No genetic variants of monogenic inflammatory bowel diseases were detected on whole-exome sequencing of a peripheral blood specimen. GPA was diagnosed based on the American College of Rheumatology criteria, and the 2012 Revised International Chapel Hill Consensus Conference on the Nomenclature of Systemic Vasculitis [1] and the Endorsed Consensus Criteria for the Classification of Childhood Vasculitides of the European League Against Rheumatism (EULAR)/Paediatric Rheumatology European Society (PRES) [7], the EULAR/Paediatric Rheumatology International Trials Organisation (PRINTO)/PRES proposed validated classification criteria [8], and the algorithm of the European Medicines Agency (EMEA) [9].

**Table 1:** Laboratory findings of the patient on admission

	Laboratory data	Reference range
White blood cell count [/μL]	7200	3900–9800
Differential (%)		
Neutrophils	4900 (68.5)	1800–8000
Lymphocytes	1700 (23.5)	1200–5200
Eosinophils	140 (2.0)	< 400
Hemoglobin [g/dL]	8.3	12.6–16.5
C-reactive protein [mg/dL]	2.4	< 0.3
Erythrocyte sedimentation rate [mm/h]	93	< 10
Ferritin [ng/mL]	31	25–280
Total protein [g/dL]	7.4	6.3–7.8
Albumin [g/dL]	3.3	3.8–4.8
Creatinine [mg/dL]	0.62	0.54–1.05
IgG [mg/dL]	1901	600–1344
Brain natriuretic peptide [pg/mL]	27.5	< 18.4
PR3-ANCA [IU/mL]	141	< 3.5
Fecal human hemoglobin [ng/mL]	105	< 50

*PR3-ANCA* serine proteinase 3-anti-neutrophil cytoplasmic antibody

After diagnosis, treatment with intravenous prednisolone (PSL) (40 mg/day) and azathioprine (AZA) (100 mg/day) was started on the 21st day of hospitalization, and negative fecal occult blood was confirmed on the 29th day. The patient was discharged on the 36th day, and PR3-ANCA converted to negative during outpatient treatment. However, the PR3-ANCA level increased again, the fecal occult blood test became positive, and micro occult blood and protein were found on urinalysis immediately after PSL tapering was completed. Oral PSL was resumed, and methotrexate (MTX) (12 mg/week) was started after the recurrence**.** Currently, he has no apparent clinical symptoms, and PR3-ANCA has remained around 3.0 IU/mL with the combination therapy of AZA and MTX.

## Discussion and conclusions

GPA is classified as a subcategory of small vasculitis, anti-neutrophil cytoplasmic antibody-associated vasculitis (AAV), in the CHCC 2012. AAV also includes microscopic polyangiitis (MPA) and eosinophilic polyangiitis with polyangiitis (EGPA) [1].

In GPA, PR3-ANCA is frequently elevated, and the sensitivity and specificity of PR3-ANCA for GPA were 65–67% and 86–89%, respectively [10]. The mechanism by which ANCA is involved in GPA has been unclear. Neutrophil extracellular traps (NETs), which are chromatin fibers released by PR3-ANCA and neutrophil activation by inflammatory cytokines, vascular endothelial cell damage due to abnormal cytokine production, and neutrophil cell death were found to be collectively involved in the pathogenesis of GPA [11]. The titer of PR3-ANCA has been generally recognized as an indicator of vasculitis [12, 13]. The main symptoms of GPA were upper and lower airway tract and renal symptoms, with frequencies of almost 70%, 78%, and 65%, respectively, and increased PR3-ANCA titers and detection of histological granulomatous inflammation were seen in 63% and 54% of GPA patients, respectively [8]. In typical GPA, the main symptoms are often observed in the order of upper airway, pulmonary, and renal symptoms, whereas the present case was atypical; no main symptoms were observed with preceding cardiac and gastrointestinal symptoms, except for suspected sinus lesions found on head MRI. However, EULAR/PRINTO/PRES childhood GPA/Wegener’s granulomatosis includes the following 6 findings: (1) histopathological granulomatous inflammation; (2) upper respiratory tract lesions; (3) laryngeal, tracheal, and bronchial lesions; (4) pulmonary lesions on chest X-ray and CT; (5) positive MPO/PR3-ANCA; and (6) renal lesions. Of these, histopathological granulomatous inflammation, upper airway lesions, and positive PR3-ANCA were observed in the present case. Thus, the present case can be diagnosed as childhood GPA with sensitivity of 93.3% and specificity of 99.2% [8]. There were few past reports of GPA in which the initial symptoms were derived from cardiac and gastrointestinal complications, as in the present case. In a literature survey, no GPA cases of cardiac tamponade due to pericarditis have been reported previously. Only one adult EGPA case (not a GPA case) with cardiac tamponade was reported [14]. In the present literature survey, 21 GPA cases resulting in bloody stool were identified, most commonly in middle-aged males with bleeding sources in the small and large intestine, and most appeared synchronously or were delayed with respect to airway and renal symptoms. In these cases, gastrointestinal symptoms preceded in only three adult cases, and the source of bleeding in all cases was the colon. In addition, two of these three adult cases rapidly developed renal dysfunction and alveolar hemorrhage later [15–17]. An additional table shows this in more detail (see Additional file 1).

Ledo et al. recently reported an 18-year-old boy with GPA from Hungary who had abdominal pain, vomiting, and diarrhea as his initial presentation, and they provided a literature summary of four recent cases of gastrointestinal GPA. None of these cases was younger than the present case, and none presented with cardiac complications [18].

The characteristics of adult and pediatric cases are clearly different. A comparison of adult and pediatric cohorts by Fina et al. [19] showed that pediatric cases have a significantly higher incidence of otolaryngological symptoms (86%), cardiac complications (57%), especially pericarditis (50%), cutaneous lesions (71%), and gastrointestinal lesions (64%).

In pediatric cases, pulmonary complications are extremely frequent (100%) [19], and care should be taken to differentiate them from refractory asthma. Pathological examination is necessary for differential diagnosis from other types of vasculitis. However, proof of pathological vasculitis can be obtained in significantly fewer cases (17%) than adult counterparts, partly because pediatric cases are often detected in the early eosinophilic phase. The recurrence rate is significantly higher in children than in adults [19].

The treatment plan for the patient in the present case was made according to the disease severity determined using the EULAR recommendation [20]. First, because the patient presented with cardiac tamponade caused by pericarditis with effusion, PSL was used for the severe form of GPA, and remission was confirmed within 2 weeks with CRP turning negative, the PR3-ANCA titer decreasing, and disappearance of bloody stool. EULAR recommends a combination of PSL and cyclophosphamide as first-line induction therapy for severe GPA. However, PSL monotherapy was chosen in 10 of the 14 patients as first-line treatment, and add-on immunomodulators were initially used in only 4 cases, even in the largest pediatric cohort reported to date [19]. This may reflect the lack of an established standard of care for pediatric patients.

The parents of the present case did not give their consent to the initial use of cyclophosphamide, so the patient was treated with PSL alone for induction therapy, with a fairly good response. AZA was used as the remission maintenance drug. After PSL was stopped, bloody stool and an increased PR3-ANCA titer appeared, resulting in relapse; therefore, combination therapy with MTX was also required. According to a cohort study of Japanese AAV performed by the Japan Research Committee of the Ministry of Health, Labour, and Welfare for Intractable Vasculitis, in Japanese GPA, the remission rate at 6 months after starting treatment was 87%, the relapse rate at 18 months after starting treatment was 15%, and the mortality rate at 24 months after starting treatment was 6% [21]. In addition, because GPA progresses rapidly, aggressive immunosuppressive therapy was selected even for this case.

In conclusion, the present case is the first pediatric GPA case with cardiac and gastrointestinal complications preceding major symptoms, as well as a rare case of cardiac tamponade due to pericarditis, which was not reported in past GPA cases. Based on the experience of this case, serial measurement of ANCA levels is important in patients with persistent fever and bloody stool, such as in inflammatory bowel disease or other chronic gastrointestinal diseases, to make the diagnosis of a vasculitic syndrome.

## Supplementary Information

**Additional file 1:** Summary of the present case and previously reported cases with preceding gastrointestinal symptoms.

**Additional file 2:** **Figure S1.** Findings of head MRI (T1-weighted image). Bilateral thickened nasal mucosa (arrowhead) and a cystic mass in the left sphenoid sinus (arrow) are found.

## Data Availability

The datasets used in this report are available from the corresponding author on reasonable request.

## Abbreviations

AAV: Anti-neutrophil cytoplasmic antibody-associated vasculitis
AZA: Azathioprine
CHCC 2012: 2012 Chapel Hill Consensus Conference
CS: Colonoscopy
EGD: Esophagogastroduodenoscopy
EGPA: Eosinophilic granulomatosis with polyangiitis
EMEA: European Medicines Agency
EULAR: European League Against Rheumatism
GPA: Granulomatosis with polyangiitis
MPA: Microscopic polyangiitis
MTX: Methotrexate
NETs: Neutrophil extracellular traps
PRES: Paediatric Rheumatology European Society
PRINTO: Paediatric Rheumatology International Trials Organisation
PR3-ANCA: Serine proteinase 3-anti-neutrophil cytoplasmic antibody
PSL: Prednisolone
SD: Standard deviation
