# Supplementary material for: Novel pediatric granulomatosis with polyangiitis with a marked bloody pericardial effusion and bloody stool: a case report
Source: Allergy Asthma Clin Immunol. 2021 Dec 4;17:124. doi: 10.1186/s13223-021-00627-1 (PMC8645136; doi:10.1186/s13223-021-00627-1)
Supplement: Supplementary file 1 — Additional file 1: Summary of the present case and previously reported cases with preceding gastrointestinal symptoms. [file 13223_2021_627_MOESM1_ESM.docx]

| **Additional file 1** Summary of the present case and previously reported cases with preceding gastrointestinal symptoms | | | | |
| --- | --- | --- | --- | --- |
|  | **Present case** | **Case 1** | **Case 2** | **Case 3** |
| **Age (y)** | 14 | 30 | 43 | 79 |
| **Sex** | male | male | male | female |
| **Chief complaints** | bloody stool, fever | bloody stool, fever, oral aphthae | bloody stool, fever, nausea, weight loss | bloody stool |
|  |  |  |  |  |
| **Location of gastrointestinal lesion** | cecum - right transverse colon | right transverse colon | cecum - hepatic flexure | total colon |
|  |  |  |  |  |
| **Histological findings** | granulomatous inflammation | non-granulomatous inflammation | non-granulomatous inflammation | non-granulomatous inflammation |
|  |  |  |  |  |
| **Complications** | cardiac tamponade | skin ulcer | skin ulcer, renal damage, lung lesion | renal damage, alveolar bleeding |
|  |  |  |  |  |
| **PR3-ANCA** | positive | positive | positive | positive |
| **Treatments** | PSL, AZA, MTX | PSL, AZA | PSL, cyclophosphamide | PSL, cyclophosphamide, methylprednisolone pulse, plasmapheresis |
|  |  |  |  |  |
| **Remission maintenance** | good | good | good | good |
| **Reference** |  | Yoshikawa  (2017) [14] | Morchón-Simón  (2011) [15] | Qian  (2010) [16] |
| There were preceding gastrointestinal symptoms in only three adult cases, and the source of bleeding in all cases was the colon. Two of the three adult cases rapidly developed renal dysfunction and alveolar hemorrhage later. | | | | |
