# Supplementary figures and images for: Novel pediatric granulomatosis with polyangiitis with a marked bloody pericardial effusion and bloody stool: a case report
Source: Allergy Asthma Clin Immunol. 2021 Dec 4;17:124. doi: 10.1186/s13223-021-00627-1 (PMC8645136; doi:10.1186/s13223-021-00627-1)

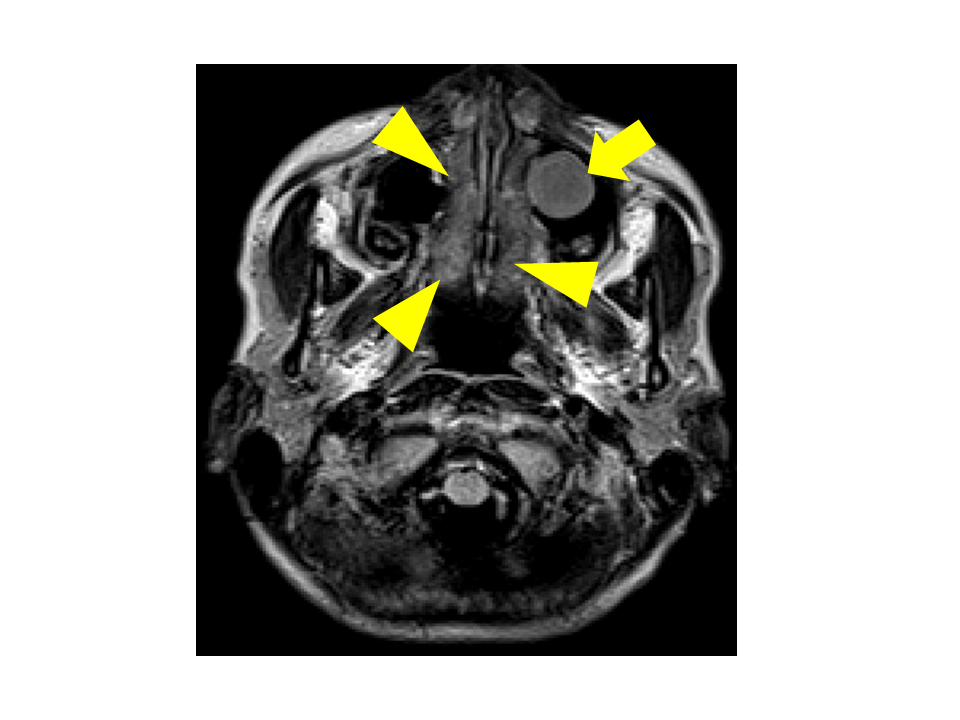

Supplement: Supplementary file 2 — Additional file 2: Figure S1. Findings of head MRI (T1-weighted image). Bilateral thickened nasal mucosa (arrowhead) and a cystic mass in the left sphenoid sinus (arrow) are found. [file 13223_2021_627_MOESM2_ESM.tif]
